# Ossification process involving the human thoracic ligamentum flavum: role of transcription factors

**DOI:** 10.1186/ar3458

**Published:** 2011-09-13

**Authors:** Kenzo Uchida, Takafumi Yayama, Hong-Xin Cai, Hideaki Nakajima, Daisuke Sugita, Alexander Rodríguez Guerrero, Shigeru Kobayashi, Ai Yoshida, Ke-Bing Chen, Hisatoshi Baba

**Affiliations:** 1 Department of Orthopaedics and Rehabilitation Medicine, Faculty of Medical Sciences, Fukui University, Eiheiji, Fukui 910-1193, Japan; 2 Department of Orthopedic Surgery, Sir Run Run Shaw Hospital, Zhejiang University School of Medicine, Hangzhou 310016, People's Republic of China; 3 Servicio de Neurocirugia, Hospital Nacional Rosales, Universidad de El Salvador, San Salvador 106-8000, El Salvador; 4 Department of Spinal Surgery, The First Affiliated Hospital, Sun Yat-Sen University School of Medicine, Guangzhou 510080, People's Republic of China

## Abstract

**Introduction:**

Ossification of the ligamentum flavum (OLF) of the spine is associated with serious neurologic compromise, but the pathomechanism of this process remains unclear. The objective of this study was to investigate the pathomechanism of the ossification process, including the roles of various transcriptional factors in the ossification of human thoracic ligamentum flavum.

**Methods:**

Sections of the thoracic ligamentum flavum were obtained from 31 patients with OLF who underwent posterior thoracic decompression, and from six control patients free of OLF. Cultured ligamentum flavum cells (*n *= 6, each) were examined with real-time reverse transcription-polymerase chain reaction (RT-PCR) analysis for Sry-type high-mobility group box 9 (Sox9), runt-related transcription factor 2 (Runx2), muscle segment homeobox 2 (Msx2), Osterix, distal-less homeobox 5 (Dlx5), and AP-1. The harvested sections were examined with hematoxylin-eosin, the terminal deoxynucleotidyl transferase-mediated dUTP-biotin nick end-labeling (TUNEL) method, and immunohistochemistry for the transcriptional factors.

**Results:**

Compared with the control, the OLF showed disorganization of the elastic fiber bundles and abundant hypertrophic chondrocytes in the ossification front. TUNEL-positive chondrocytes were found near the ossified plaques. The mRNA expression levels of Sox9, Runx2, Msx2, and AP-1 in cultured cells from the ligamentum flavum of OLF patients were significantly different from those of the control. OLF samples were strongly immunoreactive to Sox9, Runx2, and Msx2 at proliferating chondrocytes in the fibrocartilage area. Hypertrophic chondrocytes were positive for Runx2, Osterix, Dlx5, and AP-1.

**Conclusions:**

The ossification process in OLF seems to involve chondrocyte differentiation under the unique expression of transcriptional factors. Accumulation of hypertrophic chondrocytes was evident around the calcified area at the ossification front, and we suggest that the differentiation of these cells seems to be concerned with the ossification process.

## Introduction

Various pathologic conditions are known to result in ossification of the spinal ligament with subsequent neurologic compromise. Ossification of the ligamentum flavum (OLF) is an isolated form of spinal column ossification but also occurs in association with diffuse idiopathic skeletal hyperostosis, ankylosing spondylitis, and metabolic diseases such as Paget disease, hypoparathyroidism, and X-linked hypophosphatemia [1-5]. This clinical entity has been reported almost exclusively in northern East Asian countries, although it has been investigated in other regions, including southern China, India, the Middle East, and Caribbean Islands [6-8]. Although recent advances in radiologic and electrophysiological techniques allow early diagnosis of OLF [9,10], little is known about the spatial progress within the spinal canal, and no standardized treatment is known for OLF.

Several studies have described the possible roles of mechanical, genetic, metabolic, and cell biologic factors in the development and progression of OLF [11-15]. From a histopathologic point of view, enchondral ossification contributes to bone formation in the spinal ligaments [16]. The ossification fronts exist between the ossified plaque and the non-ossified fibrous area; they form a multiple-layer structure that includes the fibrocartilage layer, calcification front, calcified-cartilage layer, and ossified region. In our previous studies, we observed disordered orientation of the elastic fibers and expansion of the cartilaginous area in the early stages of ossification in the *twy/twy *mouse, which is known to develop spontaneous spinal ligament ossification [11,17]. In human samples of the ossified spinal ligament, the ossification front includes proliferating small blood vessels and clusters of hypertrophic chondrocytes producing various forms of collagen (such as collagen type II or type X), particularly around the calcification front [18,19]. Thus, we considered that these chondrocytes are involved in the progression of OLF, although these pathologic processes have not yet been elucidated.

The present study is an extension of our previous studies [11,17-19] and was designed to determine the ossification process in human OLF samples. Specifically, we focused on the expression and localization of the transcriptional factors that modulate the properties of fibroblasts-like cells and chondrocytes at the ossification front during the process of ossification.

## Materials and methods

### Patient population

A total of 31 patients who presented with progressive symptoms and signs of myelopathy and radiologic evidence of OLF (18 men, 13 women; mean age at surgery, 69.0 years; range, 52 to 86 years) underwent posterior decompressive surgery for thoracic OLF between 2001 and 2009. Samples of non-ossified ligamentum flavum obtained from six patients (three men and three women; mean age, 69.8 years; range, 60 to 81 years), who underwent posterior surgery for fracture or disc herniation in the thoracic spine, served as the controls. None of the patients had evidence of congenital bone or joint disorders or musculoligamentous tissue abnormalities. None of the patients was positive for rheumatoid factor, had hyperparathyroidism, or was taking glucocorticoids or bisphosphonate.

**Figure 1:**
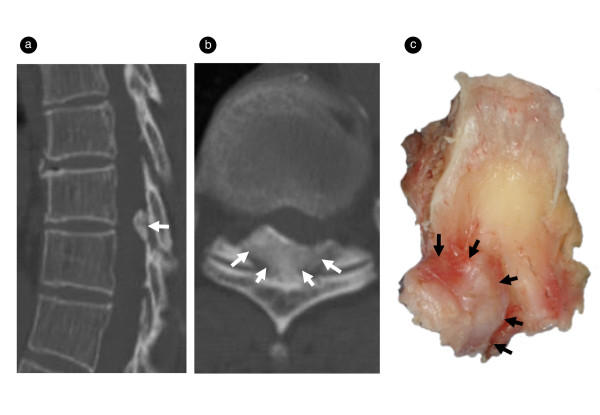
**Representative case of tuberous type OLF**. Sagittal **(a) **and axial **(b) **CT images obtained in a 72-year-old woman with T11-12 OLF. Note the fusing of the ossified plaque at midline and the progression of this process into the spinal canal. The resected sample **(c) **contains a hard mass from dura mater aspect (arrow, ossification). CT, computed tomography; OLF, ossification of the ligamentum flavum.

Patients had a variable degree of OLF-related spinal-cord impingement posteriorly and posterolaterally (Figure 1a, b). The lesion was categorized as lateral (six cases), extended (seven cases), enlarged (seven cases), fused (six cases), or tuberous (five cases) subtype, based on examination of computed tomography (CT) images taken at the level suspected to be responsible for the myelopathy and the proposed CT classification of thoracic OLF [20,21].

All patients underwent posterior decompressive surgeries: one-level decompression in 19 patients, two-level decompression in 10 patients, and three-level decompression in two patients. A level-specific diagnosis was made based on neurologic signs, CT findings, spinal cord-evoked potentials, and magnetic resonance imaging (MRI: 1.5-Tesla Signa, General Electric, Milwaukee, WI, USA).

The applied surgical technique was described in detail in our previous publication [19]. The OLF sample was obtained through a posterior midline approach followed by exposure of the vertebral laminae and ligamentum flavum. The spinous processes and cortices of the laminae near the OLF lesion were cut or resected, by using either a high-speed surgical drill or a micro-Kerrison rongeur (with a 2-mm blade and 1-mm thickness), under a direct surgical microscope (OPM 16CFC; Carl-Zeiss, Oberkochen, Germany). Because the OLF lesion often extended laterally, just medial to the facet joint, approximately the medial one third of the facet joint at the affected vertebral level(s) was resected, followed by cutting the inner cortex of the far-lateral site of the lamina. The OLF lesion was then isolated and floated like an "island" on the dura mater *vis a tergo*. At that stage, a pair of epidural electrodes (Unique Medical, Tokyo, Japan) was positioned approximately 4 to 6 cm away from the OLF lesion, proximally and distally, for spinal cord monitoring [9]. A pair of fine-tip skin-hooks was then applied to the sides of OLF lesion to lift carefully the ossified plaque dorsally away from the dura mater, before it was finally resected. The dura mater was also sometimes resected even when ossified, but care was taken to preserve the arachnoid membrane.

A written informed consent was obtained from each patient, and the study protocol strictly followed the Human Ethics Review Committee of our University.

### Cell culture

Ligament specimens were harvested under aseptic conditions during surgery from six patients (three men and three women; mean age at surgery, 71.0 years; range, 59 to 84 years; four fused type and two tuberous type on CT classification) for cell culture. Samples of non-ossified ligamentum flavum (three men, three women; mean age, 69.8 years; range, 60 to 81 years) were obtained from six patients who underwent thoracic posterior surgeries and served as the control. The tissues surrounding the ligaments were removed carefully under a dissecting microscope, and the ligaments were extirpated carefully from the not-ossified areas to avoid contamination with osteogenic cells. The collected ligaments were minced into approximately 0.5-mm^3 ^pieces and then plated onto 25-mm^2 ^culture dishes and maintained in Dulbecco modified Eagle medium supplemented with 10% fetal bovine serum, 100 unit/ml penicillin G sodium, and 100 μg/ml streptomycin sulfate in a humidified atmosphere of 95% air and 5% CO_2 _at 37°C. At confluence, the cells were harvested from the dishes with 0.02% ethylenediaminetetraacetic acid (EDTA) and 0.05% trypsin for further passages. The cultured cells were characterized at the third passage. Samples of six control specimens were cultured in a similar fashion.

### Real-time reverse transcription-polymerase chain reaction (RT-PCR) analysis

Real-time RT-PCR was used for semiquantitative analysis of the relative mRNA expression levels of transcriptional factors (repeated 3 times for each sample), by using cultured ligamentum flavum cells from six ossified samples and six control non-ossified samples. The cultured cells on each plate were disrupted in a lysis buffer containing β-mercaptoethanol, and the total RNA was purified by using RNAiso Plus (code no. 9108/9109; Takara Biomedicals, Ohtsu, Japan) and treated with DNase I (Takara Biomedicals). Reverse transcription was performed by using 1 μg of total RNA, PrimeScript RT reagent Kit (RR037A; Takara Biomedicals) and a random primer. Real-time RT-PCR was performed on SYBR Premix Ex Taq II (RR081A, Takara Biomedicals) by using 1 μl of the synthesized cDNA and SYBR Green PCR master mix (PE Applied Biosystems, Foster City, CA). The primer sequences used in the present study were Sox9, 5'-ACC AGT ACC CGC ACT TGC AC-3' and 5'-CGC TTC TCG CTC TCG TTC AG-3'; Runx2, 5'-CAC TGG CGC TGC AAC AAG A-3' and 5'-CAT TCC GGA GCT CAG CAG AAT AA-3'; Msx2, 5'-ATG CCA CGC CAG TGG GAT A-3' and 5'-TGC ACG CAG GGT TAG CAG AG-3'; Osterix, 5'-GCC ATT CTG GGC TTG GGT ATC-3' and 5'-GAA GCC GGA GTG CAG GTA TCA-3'; Dlx5, 5'-TAG CTA CGC TAG CTC CTA CCA CCA G-3' and 5'-GGT TTG CCA TTC ACC ATT CTC A-3'; and AP-1, 5'-GGG AAC AGG TGG CAC AGC TTA-3' and 5'-GCA ACT GCT GCG TTA GCA TGA-3'. The target genes were amplified and analyzed in triplicate by using ABI Prism 7000 SDS software. The expression levels of the target genes were estimated relative to that of glyceraldehyde-3-phosphate dehydrogenase (GAPDH).

All data were expressed as mean ± SEM. Differences between groups were examined with the Student *t *test. A *P *value < 5% was considered significant.

### Histopathologic processing and immunohistochemical staining

Histopathologic and immunohistochemical examinations were performed as described previously by our group [17-19,22]. The resected OLF plaque together with the surrounding ligament and ligamentous enthesis were bisected midsagittally and then fixed with 10% buffered formaldehyde for 48 hours at 4°C, and further decalcified for 4 to 7 days at 4°C in 0.5 *M *EDTA; and 0.5 *M *Tris-HCl buffer at pH 7.6, and then embedded in paraffin by using standard procedures. Serial 4-μm-thick sagittal sections of the OLF-ligament-enthesis complex were prepared for hematoxylin and eosin (H&E) and elastica van Gieson (EVG) staining.

For immunohistochemical staining, serial 4-μm-thick sections were prepared from the paraffin-embedded specimens, deparaffinized with xylene, and treated with ethanol. After washing with water, the intrinsic peroxidase was blocked with 0.3% H_2_O_2_-methanol solution at 20°C for 10 minutes and washed with phosphate-buffered saline (PBS; pH 7.4). The sections were irradiated 3 times in a polypropylene side-holder with a cap filled with PBS over a period of 5 minutes, by using a microwave oven (500 W, ER-245; Toshiba, Tokyo). The sections were then reacted with blocking solution (PBS containing carrier protein and 15 mM sodium azide LSAB kit (Lot 00075; Dako, Glostrup, Denmark) at 20°C for 10 minutes. This was followed by reaction with the following primary antibodies at 4°C overnight: rabbit polyclonal anti-Sox9 (DO406; Santa Cruz Biotechnology, Santa Cruz, CA, containing 200 μg IgG in 1 ml PBS with < 0.1% sodium azide and 0.1% gelatin), mouse monoclonal anti-Runx2 (JRH02; R&D Systems, Minneapolis, MN; containing 0.2 μm filtered solution in PBS with 5% trehalose), rabbit polyclonal anti-Msx2 (CO404; Santa Cruz Biotechnology; containing 200 μg IgG in 1 ml PBS with < 0.1% sodium azide and 0.1% gelatin), rabbit polyclonal anti-Osterix (20450; GeneTex, San Antonio, TX; containing 1 mg IgG in 1 ml PBS with 2% sucrose), rabbit polyclonal anti-Dlx5 (1; Proteintech Group, Chicago, IL; liquid solution, PBS with 0.1% sodium azide and 50% glycerol), and rabbit polyclonal anti-AP-1 (SH0302281; Abgent, San Diego, CA; containing 0.25 mg/ml IgG in 0.4 ml PBS with 0.09% NaN3) antibodies. Sections were further reacted with goat anti-mouse immunoglobulin antibodies and conjugated to peroxidase labeled-dextran polymer (EnVision; peroxidase, mouse, Dako) at 20°C for 45 minutes and then rinsed with PBS at pH 7.4. To visualize the peroxidase color reaction, sections were incubated with DAB-HCl solution (CB090; Dojin Chemicals, Tokyo; 50 mg dissolved in 100 ml of 0.05 *M *TRIS-HCl buffer at pH 7.4) at 20°C for 10 minutes, and then washed in water. Nuclear counterstaining was carried out with hematoxylin.

### Terminal deoxynucleotidyl transferase-mediated dUTP-biotin nick-end labeling (TUNEL) staining

Apoptotic cell death was assessed with the TUNEL technique. The specimens were deparaffinized and dehydrated by using standard protocols; the tissue sections (4 μm) were incubated with blocking solution (0.3% H_2_O_2 _in methanol) for 30 minutes at room temperature. After rinsing with PBS (pH 7.2), the sections were incubated for 15 minutes at 37°C with proteinase K solution (10 μg/ml in 10 mM Tris-HCl buffer, pH 7.4) and rinsed twice with PBS. TUNEL reaction mixture (50 μl enzyme solution (TdT from calf thymus in storage buffer) added to 450 μl labeled solution (nucleotide mixture in reaction buffer) and mixed well to equilibrate components) was prepared immediately before use, placed on slides (50 μl/slide), and incubated for 60 minutes at 37°C. For the negative control, the labeled solution without terminal transferase was placed on slides (50 μl/slide) instead of the TUNEL reaction mixture. These were rinsed 3 times with PBS, added to DAB substrate (10 μl 30% H_2_O_2 _added to 5 mg/ml DAB in 50 mM Tris-HCl buffer, pH 7.4) and incubated for 10 minutes at room temperature. The slides were rinsed with distilled water and counterstained with methyl green (1%) for 5 minutes. After mounting, the specimens were analyzed under light microscopy.

### Transmission electron microscopy

The ligamentum flavum tissue was resected by trimming (1.0 mm^3^) and then fixed in 2.5% glutaraldehyde water solution for 2 hours. It was dehydrated with propylene oxide (Nakalai, Kyoto, Japan), and embedded in Epon 812 (Oken, Tokyo). In the final stage, 100-nm-thick ultrathin sections were prepared by using an ultramicrotome (Ultracut N; Reihert, Wien, Austria), and stained with 2% uranyl acetate (50% ethanol solution), and observed under a transmission electron microscope (H-700; Hitachi, Tokyo).

## Results

### Histopathology, TEM, and TUNEL findings

Macroscopically, the OLF showed round, hard protrusions with a relatively smooth surface at certain regions of the dorsal surface (Figure 1c). The sagittal view showed ossification plaque that extended from the edge of the lower lamina to the upper lamina, without a clear border between the two areas, projecting ventrally into the spinal canal, accompanied by ossification of the dura mater in three (9%) patients.

**Figure 2:**
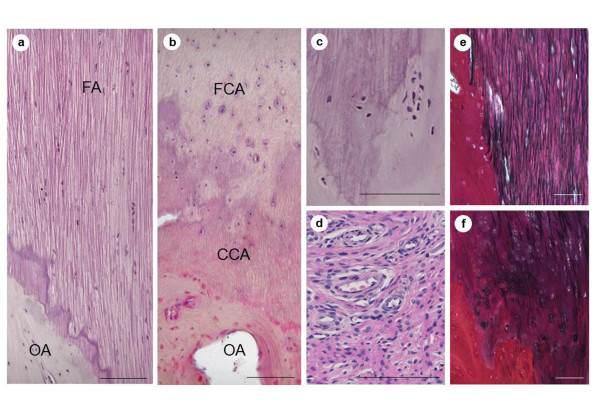
**Histologic examination of the ossification front**. Section of the control ligamentum flavum (**a**) showing a thin ossification front and regular arrangement of fiber bundles. In OLF samples (representative case of fused type), note the expanding ossification front, gathering many chondrocytes, and featuring an irregular of calcification front **(b)**. In addition to the ossification front, note the presence of osteoblasts as well as small blood cells with mesenchymal cells **(c, d)**. In the EVG image, elastic fibers exhibit a regular arrangement **(e)**, whereas the OLF samples contained irregular, fragmented fibers or no elastic fibers **(f)**. **(a-d) **H&E; **(e, f) **EVG staining. CCA, calcified cartilage area; EVG, Elastica van Gieson; FA, fiber area; FCA, fibrocartilage area; OA, ossified area; OLF, ossification of the ligamentum flavum. Scale bar = 100 μm.

Microscopically, the control samples contained a thin-layered structure between the fiber and the laminar bone, with uniform arrangement of the fibers (Figure 2a). In OLF samples, the ossification fronts included the fibrocartilage layer, calcification front, and the calcified cartilage layer, where significant irregularities and several disruptions were prominent attributes (Figure 2b). This irregularity extended not only along the longitudinal direction of the fibers but also in other directions. In addition, a significant number of chondrocytes were present around the calcification front. Around the ossification front, osteoblasts were found, just near the ossified layer, and a gathering of infiltrative small blood vessels with mesenchymal cells (Figure 2c, d). Although elastic fibers had a regular arrangement in control samples, they showed irregular arrangement, abnormally small diameter of fragmented elastic fibers, and abundant thick bundles of collagen fibers in OLF samples (Figure 2e, f). These histopathologic findings were observed to a greater or lesser degree in all patients, being more severe in larger ossified plaques (especially in the enlarged, fused, and tuberous subtypes).

**Figure 3:**
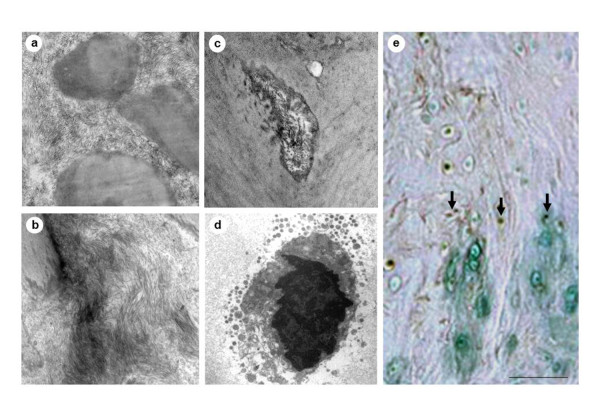
**TEM examination and TUNEL staining of the ossification front**. The typical structure of the elastic fibers had disappeared with an increase in the number of collagen fibers **(a: **control, **b: **OLF). Note the presence of matrix vesicles containing electron-dense material **(c) **and also the presence of pigmented chondrocytes **(d) **near the ossified layer **(a, b**: magnification ×1,000, **c**; magnification ×4,000, **d**; magnification ×2000). Note the presence of TUNEL-positive chondrocytes near the ossified layer (arrow) **(e**, scale bar = 100 μm). OLF: ossification of the ligamentum flavum; TEM: transmission electron microscopy; TUNEL: terminal deoxynucleotidyl transferase-mediated dUTP-biotin nick end-labeling.

Under TEM examination, OLF samples showed the disappearance of the elastic fiber structures as well as an increase in fibrotic tissue, compared with the controls (Figure 3a, b). Matrix vesicles containing electron-dense material were also seen near the chondrocytes (Figure 3c). TUNEL-positive chondrocytes with pigmented and condensed nuclei were found around the ossification plaque, representing apoptotic cells (Figure 3d, e). In particular, TUNEL-positive chondrocytes were mostly seen in the areas close to the calcification front, especially in large ossified plaques, such as the fused and tuberous types.

### Cultured cells and real-time RT-PCR analysis

**Figure 4:**
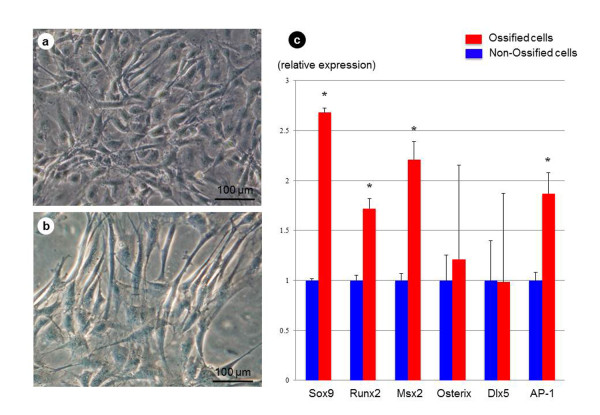
**Morphologic findings of cultured cells and relative expression levels of mRNA of transcriptional factors**. Cultured cells in OLF **(a) **and control **(b) **showed spindle shape (scale bar = 100 μm). RT-PCR analysis of the ossified and nonossified cultured ligament cells showed that the mRNA expression levels of Sox9, Runx2, Msx2, and AP-1 in OLF were significantly different from those of the control **(c)**. Data are expressed as mean ± SEM values of six samples each. Differences between the two groups were examined with the Student *t *test. *P < 0.05, compared with the control. mRNA, messenger RNA; OLF, ossification of the ligamentum flavum; RT-PCR, reverse transcription-polymerase chain reaction.

A portion of the cells from the OLF samples showed spindle-shaped morphology, being arranged in several layers. In the control samples, cultured cells also showed spindle-like morphology (Figure 4a, b).

Figure 4c shows the relative mRNA expression levels of six transcriptional factors in cultured ligamentum flavum cells by using real-time RT-PCR analysis. The mRNA expression levels (relative to the control sample) in the OLF sample were 2.7 ± 0.05 for Sox9, 1.7 ± 0.1 for Runx2, 2.2 ± 0.2 for Msx2, 1.2 ± 0.9 for Osterix, 1.0 ± 0.9 for Dlx5, and 1.9 ± 0.2 for AP-1. The relative mRNA expression levels of Sox9, Runx2, Msx2, and AP-1 in OLF samples were significantly higher than those in the control group (*P *< 0.05, each). However, the mRNA expression levels of Osterix and Dlx5 were not significantly different from the control.

### Immunohistochemical findings

**Figure 5:**
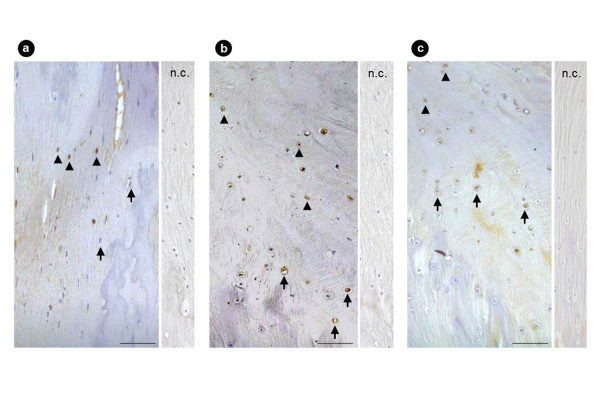
**Immunostaining for Sox9, Runx2, and Msx2 of the OLF**. Sox9 **(a) **was expressed in proliferating chondrocytes. Runx2 **(b) **was expressed in hypertrophic chondrocytes. Msx2 **(c) **was expressed in proliferating chondrocytes (scale bar = 100 μm; n.c., negative control; black arrow, hypertrophic chondrocytes; black arrowheads, proliferating chondrocytes).

**Figure 6:**
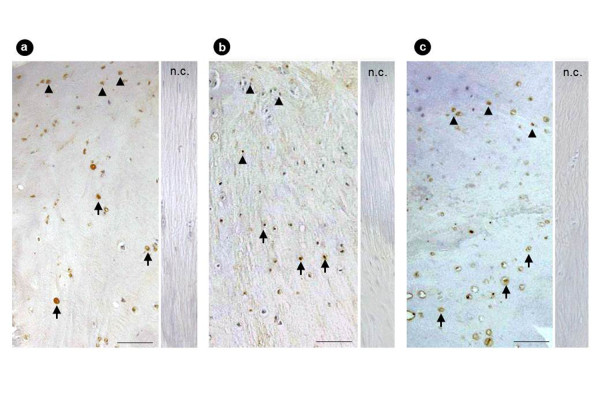
**Immunostaining for Osterix, Dlx5, and AP-1 in the OLF**. Osterix **(a)**, Dlx5 **(b)**, and AP-1 **(c) **were strongly positive in hypertrophic chondrocytes (scale bar = 100 μm; n.c., negative control; black arrow, hypertrophic chondrocytes; black arrowheads, proliferating chondrocytes). OLF, ossification of the ligamentum flavum.

In 23 of 31 OLF patients, the chondrocytes present in the ossification front were immunopositive for Sox9; the immunoreactivity was strictly limited to proliferating chondrocytes in the fibrocartilage area, whereas the hypertrophic chondrocytes close to the calcification front were negative for Sox9 (Figure 5a). Conversely, Sox9 expression was significant in mesenchymal cells present around the ossification front, where the ligamentous matrix showed degenerative changes in these patients. Runx2 expression was evident in 20 of 31 cases and localized in proliferating chondrocytes and hypertrophic chondrocytes (Figure 5b). Immunopositivity for Msx2 was noted in 24 of 31 cases and localized in proliferating chondrocytes, and in particular, the mesenchymal cells near the ossification front were strongly positive for Msx2 (Figure 5c). Positive immunostaining for Osterix was noted in 16 of 31 cases and localized in chondrocytes present in the calcified cartilage layer and fibrocartilage layer. Furthermore, numerous Osterix-positive hypertrophic chondrocytes were seen around the calcification front (Figure 6a). Immunostaining for Dlx5 was significant in 12 of 31 cases, and that for AP-1 was in 14 of 31 cases in hypertrophic chondrocytes in the calcified cartilage area (Figure 6b, c). The immunohistochemical expression of these factors is summarized in Additional files 1 and 2.

## Discussion

Ligamentum flavum is a longitudinally arranged two-layered structure lining the posterior aspect of the spinal canal and functionally allows flexibility and stabilization of the spine [23]. The normal ligament is highly elastic, based on the large proportion of elastic fibers, accounting for 60% to 70% of the dry weight [24], with little blood flow. However, the cross-sectional area and viscoelasticity of the elastic fibers diminish as part of age-related changes or systemic hormonal imbalance, although the elastic fiber bundles retain their normal longitudinal arrangement [25-27]. The pathologic process of degeneration that affects the ligamentum flavum is characterized by disorganization of the elastic fibers with irregular arrangement, a decrease in the number of these fibers, and an increase in collagen fibers [28-30].

In the ossification front of OLF, fiber bundles of the ligament showed disorganization of the elastic fibers and an abundance of collagen fibers. We reported previously that the initial degenerative changes in the ligamentum flavum include reduction in the diameter of elastic fibers together with irregular arrangement and their rupture [25]. The elastic fiber bundles showed gradual fragmentation and extinction, probably because of the action of proteases such as elastase and chymotrypsin [31]. We consider that such matrix changes are important in the development of the ossified plaque, particularly in the fused and tuberous subtypes of OLF, compared with the lateral, extended, and enlarged subtypes [19].

Previous studies examined various aspects of the OLF. Genetic studies identified abnormalities in *COL6A2 *and *COL11A1 *genes, on chromosome 21, in patients with ossification of the spinal ligaments [14,32]. The expressions of these genes may explain why this pathologic process occurs mainly in East Asian countries. Conversely, the ligamentum flavum is persistently subjected to repetitive variable degrees of tensile stress along its longitudinal axis, and mechanical stress influences the cell biologic properties, such as expression of cytokines and/or transcriptional factors [12,33,34]. Our results showed overexpression of Sox9, Runx2, Msx2, and AP-1 mRNAs in cells cultured from OLF compared with the control samples. Conversely, the mRNA expression levels of Osterix and Dlx5 were not different from the control, although the protein expression of all these factors was positive at the cells gathering in the ossification front *in vivo *environment. Considered together with the previously mentioned studies, we speculate that the observed changes are due to the diversity of genetic background as well as spinal mechanical stress, degenerative matrix changes, and/or other yet-unknown factors that influence the expression levels of these transcriptional factors.

Several studies [16,35,36] concluded that the process of ossification of the spinal ligament involves enchondral ossification and clustering of abnormal fibrocartilage or cartilaginous cells, which results in the development of the ossified plaque. Numerous chondrocytes were present, especially around the calcification front, although no such chondrocytes were found in the normal ligamentum flavum [17,25,31]. Yahia *et al*. [37] suggested that the metaplastic chondrocytes in the ligamentum flavum are derived from mesenchymal cells or fibroblast-like cells. In this regard, our study indicates the involvement of certain transcriptional factors in this process. Sox9 is known to promote chondrocyte differentiation, but to prevent hypertrophic changes [38]. Runx2 promotes the differentiation of premature chondrocytes to hypertrophic chondrocytes [39]. In contrast, Msx2 induces differentiation of premature mesenchymal cells and sometimes prevents the maturation of chondrocytes [40]. In the normal ligamentum flavum, these factors might regulate chondrocyte differentiation, preserving homeostasis; however, overexpression of these factors in the OLF violates the regulation of chondrogenesis and differentiation of mesenchymal or fibroblast-like cells to mature chondrocytes in a complex autocrine/paracrine manner.

The maturation of osteoblastic cells and bone formation correlate closely with stromal expansion and neovascularization. We reported previously that hypertrophic chondrocytes around the calcification front regulate angiogenesis by secreting various growth factors, such as vascular endothelial growth factor [19]. Neovascularization promotes infiltration of mesenchymal cells and alters matrix mineralization, particularly in the area of the ossification front. However, many TUNEL-positive hypertrophic chondrocytes were noted in the ossified front, especially around the calcification front. It is not clear whether the chemical change in the matrix induces apoptosis of the hypertrophic chondrocytes or if these cells themselves undergo programmed cell death triggered by biochemical alteration of the chondroid matrix secondary to the degeneration of the ligamentum flavum, or whether apoptosis was induced by the ossified plaque and/or matrix vesicles. We suggest that apoptosis of the hypertrophic chondrocytes induces secondary infiltration of osteoblastic cells through the expression of transcriptional factors such as Runx2, Osterix, Dlx5, and AP-1 [41-43].

## Conclusions

Our study showed significant matrix degeneration in the ossification front of OLF and the presence of several ossification fronts between the ossified plaque and non-ossified fiber area. Chondrocyte differentiation was evident in the ossification front and while accumulation of chondrocytes was evident around the calcification front. We suspect that chondrocyte differentiation under the influence of transcriptional factors plays a key role in the ossification process.

## Abbreviations

CCA: calcified cartilage area; CT: computed tomography; Dlx5: distal-less homeobox 5; EDTA: ethylene diaminetetraacetic acid; EVG: Elastica van Gieson; FA: fiber area; FCA: fibrocartilage area; GAPDH: glyceraldehyde-3-phosphate dehydrogenase; H&E: hematoxylin and eosin; MRI: magnetic resonance imaging; Msx2: muscle segment homeobox 2; n.c.: negative control; OA: ossified area; OLF: ossification of the ligamentum flavum; PBS: phosphate-buffered saline; RT-PCR: reverse transcription-polymerase chain reaction; Runx2: runt-related transcription factor 2; Sox9: Sry-type high-mobility group box 9; TEM: transmission electron microscopy; TUNEL: terminal deoxynucleotidyl transferase-mediated dUTP-biotin nick-end labeling.

## Competing interests

The authors declare that they have no competing interests.

## Authors' contributions

HB, who did not participate in the histopathologic and immunohistochemical assessments, performed the surgeries. KU and TY (principal authors), together with HC, DS, AG, AY, and KC, were blinded to the surgical findings and were responsible for all histopathologic investigations. HN and SK examined all data. All authors participated in the elaboration of this document, approving the final version of this manuscript for publication.

## Supplementary Material

Additional file 1 **Topographic analysis of the expression of transcriptional factors**. The table shows the summary of immunohistochemical localization of Sox9, Runx2, Msx2, Osterix, Dlx5, and AP-1 in all 31 cases. The tabulated data represent the distribution of immunopositive areas in the ossification front, based on semiquantitative analysis conducted according to the method described by Kokubo *et al*. [22] and Song *et al*. [35]. Click here for file

Additional file 2 **Relation between CT subtype and immunopositivity to each transcriptional factor**. The results indicate that the expression of transcriptional factors varied according to the size of the ossified plaque subtype. Immunopositivity for Sox9, Runx2, and Msx2 tended to be more common in the fused and tuberous subtypes (near 100%) than in the lateral and extended subtypes (< 50%). Expression of Osterix, Dlx5, and AP-1 was also high in the fused and tuberous subtypes. Click here for file
