# Supplementary figures and images for: Ossification process involving the human thoracic ligamentum flavum: role of transcription factors
Source: Arthritis Res Ther. 2011 Sep 13;13(5):R144. doi: 10.1186/ar3458 (PMC3308072; doi:10.1186/ar3458)

**Additional File 1**


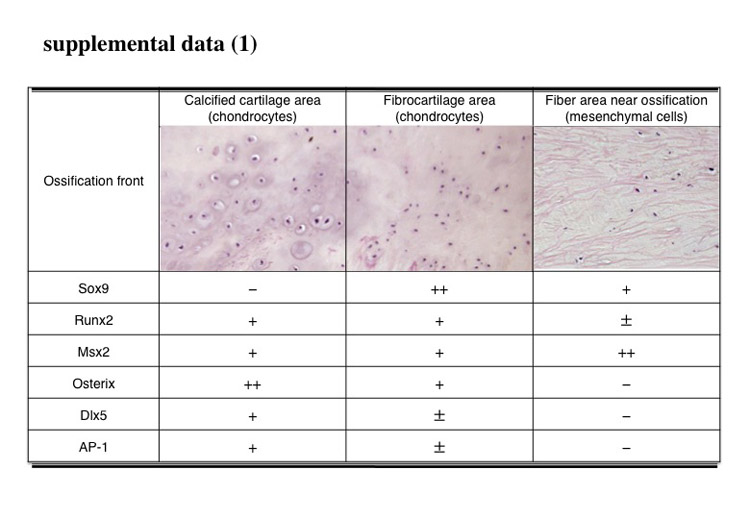


**++**, strongly positive; **+**, moderately positive; **±**, weakly positive; **–**, negative staining

Supplement: Additional file 1 — Topographic analysis of the expression of transcriptional factors. The table shows the summary of immunohistochemical localization of Sox9, Runx2, Msx2, Osterix, Dlx5, and AP-1 in all 31 cases. The tabulated data represent the distribution of immunopositive areas in the ossification front, based on semiquantitative analysis conducted according to the method described by Kokubo et al. [22] and Song et al. [35]. [file ar3458-S1.DOC]

**Additional File 2**


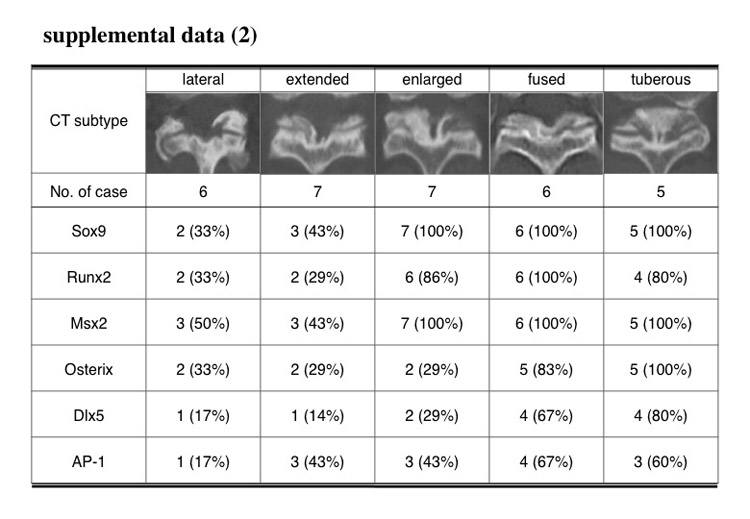

Supplement: Additional file 2 — Relation between CT subtype and immunopositivity to each transcriptional factor. The results indicate that the expression of transcriptional factors varied according to the size of the ossified plaque subtype. Immunopositivity for Sox9, Runx2, and Msx2 tended to be more common in the fused and tuberous subtypes (near 100%) than in the lateral and extended subtypes (< 50%). Expression of Osterix, Dlx5, and AP-1 was also high in the fused and tuberous subtypes. [file ar3458-S2.DOCX]
